# Responses of soccer players performing repeated maximal efforts in simulated conditions of the FIFA World Cup Qatar 2022: A holistic approach

**DOI:** 10.1371/journal.pone.0276314

**Published:** 2022-12-09

**Authors:** Paweł Chmura, Hongyou Liu, Marcin Andrzejewski, Antonio Tessitore, Jerzy Sadowski, Jan Chmura, Andrzej Rokita, Wojciech Tański, Leszek Cicirko, Marek Konefał

**Affiliations:** 1 Department of Team Games, Wrocław University of Health and Sport Sciences, Wrocław, Poland; 2 School of Physical Education & Sports Science, Guangzhou Higher Education Mega Centre, South China Normal University, Guangzhou, China; 3 Department of Methodology of Recreation, Poznań University of Physical Education, Poznań, Poland; 4 Department of Movement, Human and Health Sciences, University of Rome “Foro Italico”, Rome, Italy; 5 Department of Sport Science, Faculty of Physical Education and Health, Józef Piłsudski University of Physical Education in Warsaw, Warsaw, Poland; 6 Department of Human Motor Skills, Wrocław University of Health and Sport Sciences, Wrocław, Poland; 7 4th Military Teaching Hospital with Clinic, Independent Public Health Care Centre in Wrocław, Wrocław, Poland; 8 Department of Physical Education and Sport, Faculty of Physical Education and Health in Biała Podlaska, Józef Piłsudski University of Physical Education in Warsaw, Warsaw, Poland; Sport Sciences School of Rio Maior - Politechnic Institute of Santarem, PORTUGAL

## Abstract

This study aimed to assess the capacity for repeated maximal effort (RME) of soccer players in the thermo-natural conditions (NC) and in simulated conditions for the 2022 FIFA World Cup in Qatar (QSC). Twenty-four semi-professional soccer players participated in the study. The exercise test consisted of ten 6-second maximal efforts on a cycloergometer. A 90-second passive rest interval was used. The test was performed in a Weiss Technik WK-26 climate test chamber in two different conditions: 1) thermo-neutral conditions (NC—20.5°C; 58.7% humidity); and 2) simulated conditions for the 2022 World Cup in Qatar (QSC—28.5 ± 1.92°C; 58.7 ± 8.64% humidity). Power-related, physiological, psychomotor, blood, and electrolyte variables were recorded. Results showed that (1) players achieved higher peak power (max 1607,46 ± 192,70 [W] - 3^rd^ rep), needed less time to peak power (min 0,95 ± 0,27 [s] - 3^rd^ rep), and had a higher fatigue slope (max 218,67 ± 59,64 [W/sek] - 7^th^ rep) in QSC than in NC (in each repetition of study protocol); (2) between the 1st repetition and subsequent repetitions a number of significants in among physiological, blood-related, and electrolyte variables were noted, but their direction was similar in both simulated conditions (e.g. V’O2/kg 37,59 ± 3,96 vs 37,95 ± 3,17 [ml/min/kg] - 3^rd^ rep, LAC 13,16 ± 2,61 vs 14,18 ± 3,13 [mg/dl] - 10^th^ rep or K 4,54 ± 0,29 vs 4,79 ± 0,36 [mmol/l] - 2^nd^ rep when compare QCS and NC respectively); (3) an 8°C of temperature difference between the climatic conditions did not significantly affect the soccer players’ physical and physiological responses in RME. The study results can be used in the design of training programs aimed to increase players’ physiological adaptations by simulating soccer-specific conditions of play in terms of anaerobic capacity, in particular, repetitive maximal efforts. These findings will be useful during the upcoming 2022 World Cup in Qatar and in locations where high ambient temperatures are customary.

## Introduction

Match analysis data from the 2010 FIFA World Cup in South Africa, 2014 FIFA World Cup in Brazil, and 2018 FIFA World Cup in Russia confirm a marked tendency of increasing match performance intensity [1, 2]. In order to win in top ranked tournaments soccer players are required to cover longer total distances and distances at high intensity during matches [3, 4]. Chmura et al. [2] indicated the high demands for players in terms of speed and endurance skills necessary to compete in key phases of football games at the highest level. For example, Kołodziejczyk et al. [4] stated that players in central positions on the pitch in the knockout phase were able to maintain or even increase their high and very high intensity activity in three consecutive matches with extra time at the 2018 FIFA World Cup in Russia. This implies that elite soccer players must possess high levels of aerobic and anaerobic capacity [5].

In 2022, for the first time in history, the World Cup in Qatar will be moved to November and December to make the climatic conditions more bearable and less thermally troublesome for players [6]. According to climatologists, despite the fact that during the tournament no extremely high temperatures are expected (unlike in June and July), they can still reach or even exceed 30°C [7]. An important question is how to prepare players well for this tournament/such conditions? The effects of global warming include increasing heat-related health risks, especially in sporting events. For example, at the 2014 FIFA World Cup, Brazil’s climate became a considerable concern for many European soccer teams. Under heat stress, players’ repeated sprinting and jumping abilities are compromised to a greater extent than in temperate conditions [8]. This compromise occurs despite evidence that players adjust their physical activity patterns (e.g., decreasing total and high-intensity running distance) in the heat to maintain their capacity to perform periodic sprint efforts when required at key moments in a match [9]. Nassis et al. [10] found that athletes competing in temperatures above 28°C experienced "heat stress" and reduced high intensity activity and sprinting efforts. Similar observations were also made by Chmura et al. [11]. Therefore, it is important to study players’ responses to repeated maximal efforts, which they will be making in high temperature conditions expected in Qatar.

Repeated maximal efforts (RME) generating high power over a short distance and high running speed are the fundamental abilities of soccer players and are commonly regarded as essential performance determinants of sport success [12, 13]. Faude et al. [14] demonstrated a relationship between soccer players’ explosive performance and key moments in a soccer match. They reported that 83% of goals were preceded by actions such as short sprints or jumps [15]. Chmura et al. [2] in their analysis of the 2014 World Cup matches noted that soccer players reached the average top speed from 27.66 ± 2.32 km/h in the first match of the group stage to 28.50 ± 1.82 km/h in the final and the third place match. Several studies investigated physiological determinants and fatigue mechanisms related to this form of exercise [16]. In literature, fatigue can be defined as “an acute impairment of performance that includes both an increase in the perceived effort necessary to exert a desired force/power and eventual inability to produce this force/power” [17]. Furthering our understanding of fatigue mechanisms during RME may help to delay the onset of fatigue and improve performances [18, 19].

Ergometer RME tests in indoor settings have been commonly used when field measures are unavailable, or for the measurement of variables that are difficult to implement in the field (power, force–velocity profiling) [20]. Furthermore, cycling ergometers have been frequently applied in RSE testing as they allow rapid acceleration of pace during short exercises performed at or near players’ maximal abilities. They have also been used for conditioning athletes to specific physiological, metabolic, mechanical, and psychological demands of competition, which is a fundamental principle of athletic training [20]. Meckel et al. [21] argue that anaerobic testing procedures should consist of specific protocols that mimic the athlete’s specific sports activity pattern. The present study used a repeated sprint protocol with a 90-second interval that directly reproduces the intervals between which players make consecutive high or very high intensity efforts or sprints in top-level football matches [22, 23]. A bicycle cycloergometer was used allowing a more holistic approach to the evaluation of parameters of reactions of the player’s body, e.g. power that cannot be measured directly on the pitch. The authors have only examined some groups of parameters, e.g. variables related to power or to blood, that have usually been analyzed separately in different research projects.

Only one study to date has analyzed the mechanical and biochemical parameters in the same study protocol consisting of repeated maximal efforts (only even repetitions) in semi-professional soccer players [7]. A greater number of parameters and expanded repetitions (10) are covered in the current study. In addition, the present study not only compares the RMEs between two sets of simulated conditions, but also between the 1st repetition and subsequent repetitions. The study uses climatologists’ calculations to attempt a comparison of soccer players’ reactions to the simulated effort planned for the FIFA World Cup in Qatar 2022 and in the thermoneutral conditions for humans. The research of the impact of high ambient temperature on the ability to perform RME will contribute to a more efficient preparation of soccer players for competitions held in high-temperature locations. The present study aimed to assess soccer players’ body responses to repeated maximal efforts: 1) in thermoneutral conditions and climatic conditions predicted for the 2022 World Cup in Qatar; and 2) between subsequent effort repetitions in comparison to the 1st repetition. It has been hypothesized that performing RME at a higher temperature will support the achievement of higher maximum power, while when analyzing subsequent RME, a faster decrease in maximum power will be observed. In addition, it was expected that the responses of the players on the effort in the physiological, blood-related, and electrolyte variables will not significantly differ when comparing individual repetitions between conditions, but large changes will be observed when comparing subsequent RMEs in both conditions.

## Methods

### Participants

At the beginning, the study group comprised 28 semi-professional soccer players. During the tests, 4 players who were unable to complete the tests or who experienced medically certified conditions were excluded, which constituted 14% of the group. Finally, 24 players were qualified for the analysis. The inclusion criteria were complete test results of all players without cognitive alterations, recent surgeries or injuries. Furthermore, only outfield players were considered. All players were instructed not to consume alcohol or take any drugs for at least 24 hours before the tests, and to maintain normal diets. Tested players were aged 21.31 ± 3.63 years from a Polish 4th League club. According to The Participant Classification Framework a participants were classified as a Tier 2: Trained / Developmental [24]. The players’ mean body height was 179.77 ± 6.16 cm and mean body mass was 76.02 ± 5.64 kg. All players had 9.0 ± 1.3 years of training experience, and trained five times a week. The study was carried out in November 2018 during the 2017/2018 league season.

All participants were provided with a detailed explanation of the study purpose and requirements. They were informed of potential risks and gave their written consent to participate. Additionally, parental or guardian consent was obtained from participants under 18 years of age. All players were free to withdraw from the study at any time. The study protocol had been approved by the Research Ethics Committee Wroclaw University of Health and Sport Sciences (no. 937/17) and authors obtained informed written consent from the participants. The study complied with the Declaration of Helsinki requirements as well as all relevant health and safety procedures.

### Procedures

The study protocol comprised 10 sets of 6-second repeated efforts at maximum intensity, with 90-second rest intervals between the sets [25–28]. The tests were performed on a MONARK LT2 cycloergometer (Vansbro, Sweden). The participants were to attain maximum anaerobic power on the cycloergometer in each repetition. Toe clips and heel straps at the pedals were used for foot fixation. Strong verbal motivation was provided during each exercise. All sprints were performed from the same starting pedal position with the right crank arm positioned 45° forward to the vertical axis. The participants remained on the cycloergometer for the 90-second rest after each even sprint, during which capillary blood was drawn, or the players performed a psychomotor test (in selected repetitions, respectively). Next, the power-related, physiological, psychomotor, choice reaction time, blood-related and electrolyte-related variables were compared between the hermoneutral and conditions predicted for the 2022 World Cup in Qatar. The values at each subsequent repetition were also pair compared with those at the 1st repetition.

The tests were performed in a Weiss Technik WK-26 climatic test chamber in two different settings: 1) in thermo-neutral conditions (NC—ambient temperature of 20.5°C, relative humidity of 58.7%) [29, 30]; and 2) in climatic conditions corresponding to the average maximum ambient temperature (QSC—28.5 ± 1.92°C, relative humidity of 58.7 ± 8.64%). To predict the 2022 World Cup climatic conditions, the authors together with climatologists calculated climate data over the past 10 years (from 2008 to 2018 https://en.tutiempo.net/) on the first day of the tournament in Doha, Qatar.

Before the start of each exercise series, after entering the chamber each player spent the first 10 minutes adapting at rest to the chamber conditions [31], followed by a 5-minute warm-up on the cycloergometer reaching a heart rate of 150 bpm [32, 33]. Five minutes after warm-up completion, the actual measurements began. The tests took place from 9 am to 2 pm from Monday to Friday. The test series were separated by 7 days.

### Measured variables

Power related values were recorded with an ergometer connected to an IBM compatible computer system to enable data collection to calculate the power generated at each revolution of the hand wheel and the work performed during each individual sprint repetition (Lab-VIEW, National Instruments Corp., Austin, Texas). Previously, this procedure was used in many other studies [7, 27, 33, 34], in which the individual stages of data registration and data collection were discussed in detail. The following power-related variables were recorded: peak power (W), mean power (W), time to peak power (s), total work (J), fatigue slope (W/sec), rate of fatigue (%).

Physiological respiratory parameters were recorded using a breath-by-breath measurement system (Cosmed Quark CPET, Cosmed Srl, Italy). The flow meter and gas analyzers were calibrated before each test, according to the manufacturer’s instructions (Nieman et al., 2013) [35]. The standard mask fitting procedure was followed before, during, and after the test [36]. The studied parameters included V’E (l/min), V’O2/kg (ml/min/kg), RER, and HR (bests/min). RPE (au) and choice reaction time (ms) were also recorded. RPE was recorded using a Borg scale (0–10) [37, 38]. Psychomotor performance was assessed on the basis of CRT measurements using the APR reaction measuring instrument (UNI-PAR, Warsaw, Poland). The procedure of collecting psychomotor test data corresponded to the procedures in Chmura et al. [39].

Blood- and electrolyte-related variables were collected by qualified personnel in a professional laboratory. All safety precautions were strictly followed during blood collection. Data was collected according to the procedure described by [40]. The analysed parameters included PCO2 (mmHg), PO2 (mmHg), PH, LAC (mg/dl), GLU (mg/dl), HCT (%), NA+ (mmol/l), K+ (mmol/l), CL- (mmol/l), and CA2+ (mmol/l).

### Statistical analysis

The data were analysed as a time series of repeated measurements using a spreadsheet developed by Hopkins [41]. Firstly, the mean change values of each of the 22 parameters in each repetition, as well as in rest and post-test status from NC to QSC were evaluated. Secondly, the mean change values of power-related, physiological and psychomotor variables (Peak Power, Mean Power, Time to Peak Power, Fatigue slope, Rate of Fatigue, Total Work, V’E, V’O2/kg, RER, HR, RPE) from the 1st repetition to the 2nd to 10th repetition were assessed. Moreover, the mean change values of the Choice Reaction Time from the rest status to the 3rd, 5th, 7th, 10th repetitions were evaluated. Furthermore, the mean change values of the blood-related and electrolytes-related variables (PCO2, PO2, PH, LAC, GLU, HCT, NA+, K+, CL-, CA2+) from the rest status to the 2nd, 4th, 6th, 8th, 10th repetition and post-test status were assessed.

Changes in the means of raw values as well as their 90% compatibility intervals were assessed in standardised units. For the within-subject change from NC to QSC, the mean change values were divided by the standard deviation (SD) in NC, while for the within-subject change from the 1st repetition or rest status to the 2nd to 10th repetitions or post-test status, the mean change values were divided by the SD in the 1st repetition or in the rest-status [42, 43]. The magnitudes of the mean changes were then evaluated qualitatively using the following scale: < 0.2—trivial, 0.2–0.6—small, 0.6–1.2—moderate, 1.2–2.0—large, 2.0–4.0—very large, > 4.0—extremely large. Decisions about magnitudes accounting for the uncertainty were based on hypothesis tests for significant and trivial effects [44]. Hypotheses of significant decrease and increase in the means were rejected if their respective p values (p–and p+) were less than 0.05. If only one hypothesis was rejected, the p value for the other hypothesis corresponded to the posterior probability of magnitude of the true effect in a reference-Bayesian analysis with a minimally informative prior [45] and was interpreted using the following scale: > 0.25—possibly; > 0.75—likely; > 0.95—very likely; > 0.995—most likely [46]. If neither hypothesis was rejected, the effect is described as unclear. Only very likely and most likely effects were discussed in the study.

## Results

**Fig 1:**
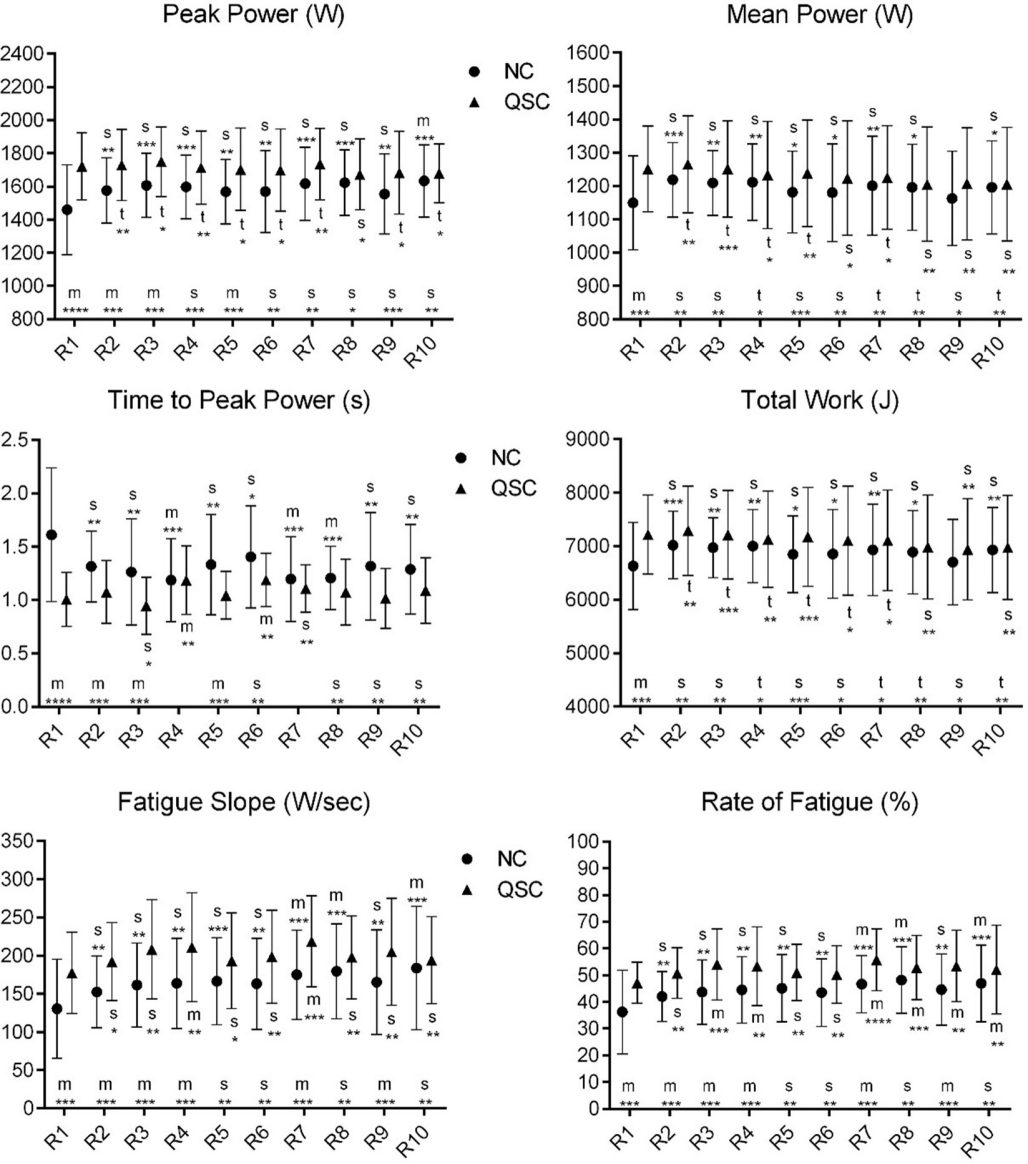
Change in the means of power-related variables in each repetition in NC and QSC. Error bars are 90% compatibility intervals. Letters stand for the magnitude of the mean change values as following: t = trivial; s = small; m = moderate. Asterisks indicate the likelihood for the magnitude of the true effect as follows: * possible; ** likely; *** very likely; **** most likely. Letters and asterisks next to the x-axis indicate the magnitude and uncertainty of the mean changes from the NC to QSC. Letters and asterisks above and below the error bars indicate the magnitude and uncertainty of the mean changes from the 1st repetition to the 2nd to 10th repetition in NC and QSC. Effects are unclear in the situations without letters or asterisks.

**Fig 2:**
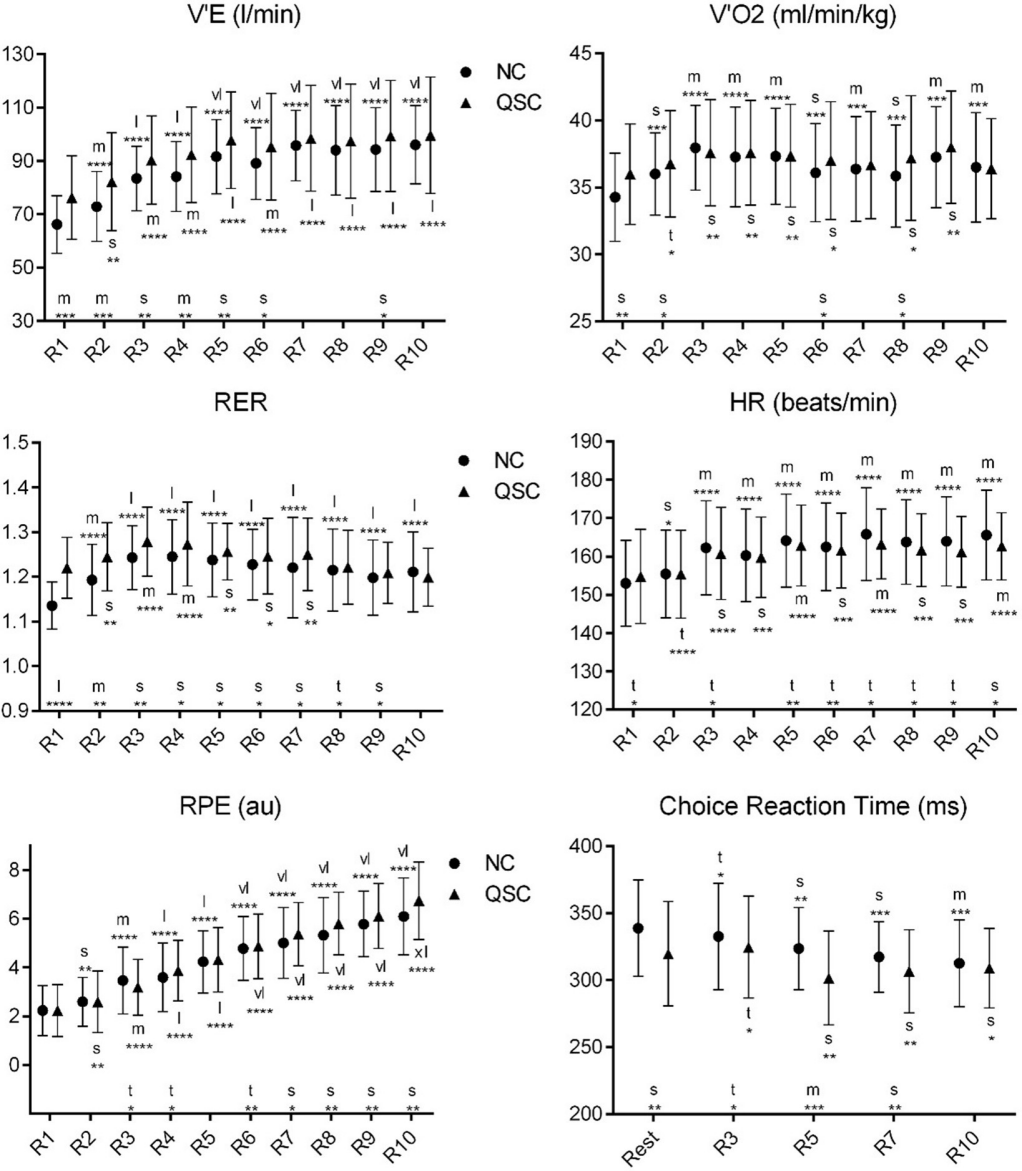
Change in the means of physiological and psychomotor variables in each repetition in NC and QSC. Error bars are 90% compatibility intervals. Letters stand for the magnitude of the mean changes as following: t = trivial; s = small; m = moderate; l = large; vl = very large; xl = extremely large. Asterisks indicate the likelihood for the magnitude of the true effect as follows: * possible; ** likely; *** very likely; **** most likely. Letters and asterisks next to the x-axis indicate the magnitude and uncertainty of the mean changes from the NC to QSC. Letters and asterisks above and below the error bars indicate the magnitude and uncertainty of the mean changes from the 1st repetition (or rest status) to the 2nd to 10th repetition in NC and QSC. Effects are unclear in the situations without letters or asterisks.

**Fig 3:**
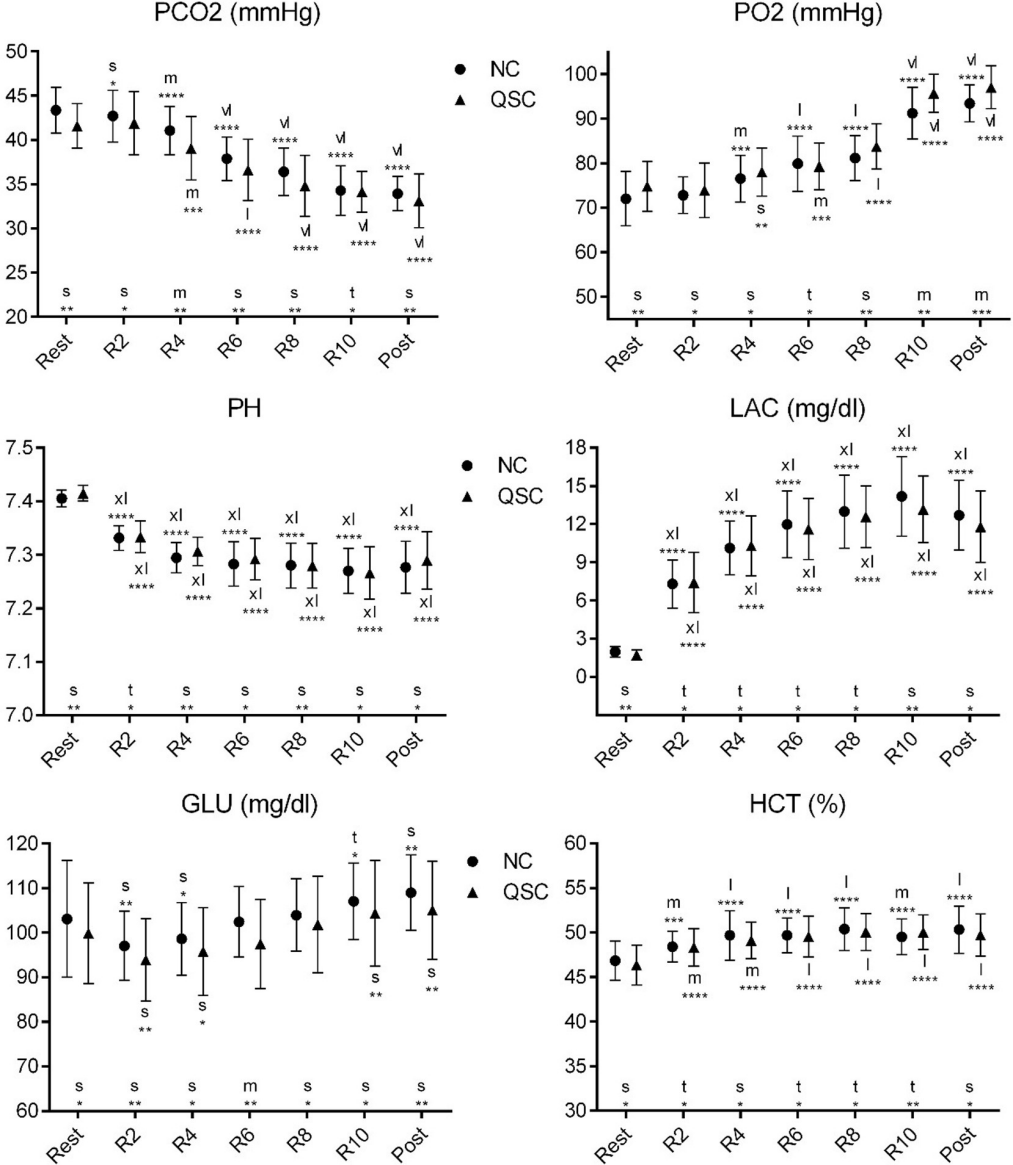
Change in the means of blood-related variables in each repetition in NC and QSC. Error bars are 90% compatibility intervals. Letters stand for the magnitude of the mean changes as following: t = trivial; s = small; m = moderate; l = large; vl = very large; xl = extremely large. Asterisks indicate the likelihood for the magnitude of the true effect as follows: * possible; ** likely; *** very likely; **** most likely. Letters and asterisks next to the x-axis indicate the magnitude and uncertainty of the mean changes from NC to the QSC. Letters and asterisks above and below the error bars indicate the magnitude and uncertainty of the mean changes from the rest status to the 2nd to 10th repetition and post-test status in NC and QSC. Effects are unclear in the situations without letters or asterisks.

**Fig 4:**
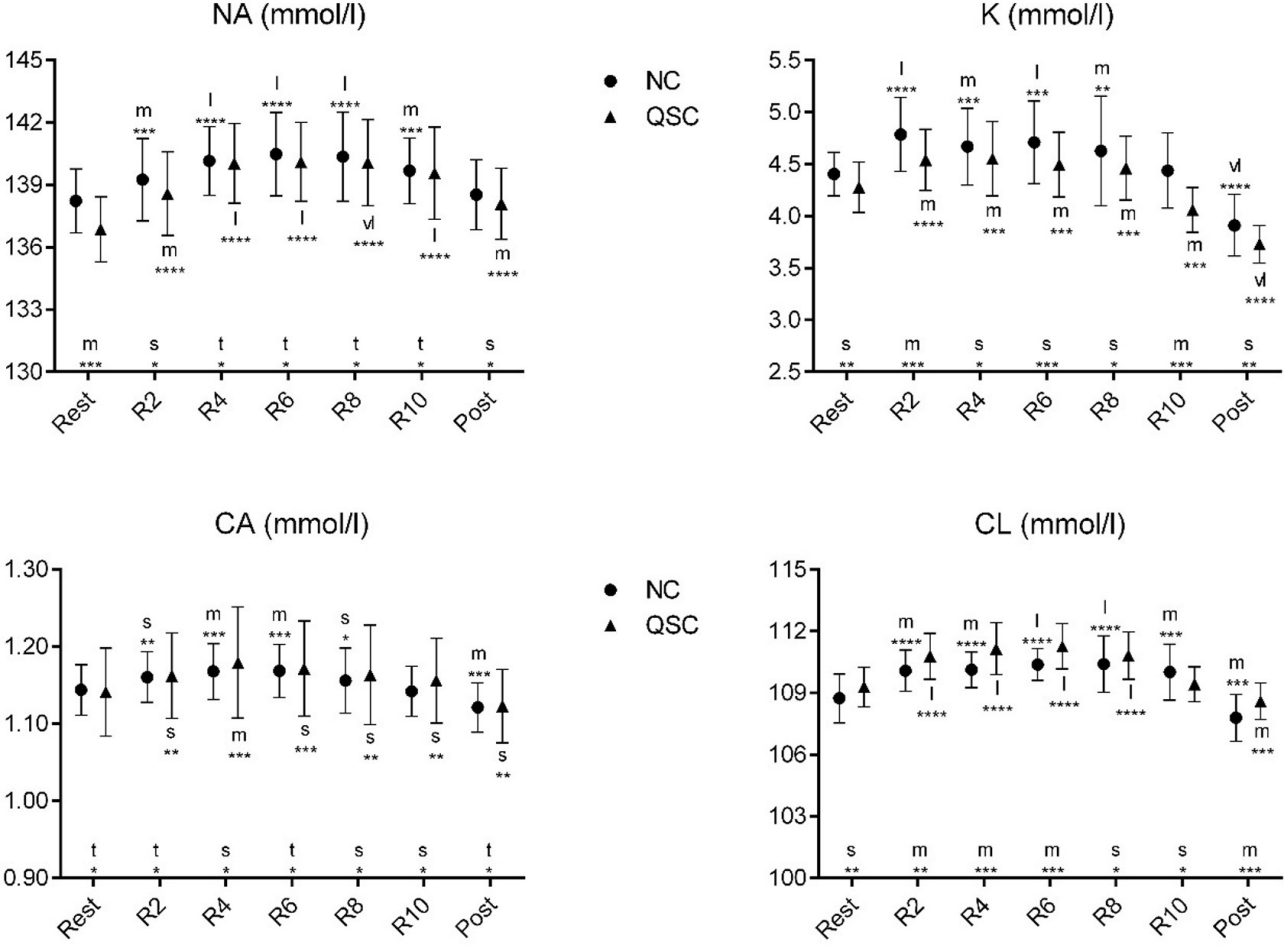
Change in the means of electrolytes-related variables in each repetition in NC and QSC. Error bars are 90% compatibility intervals. Letters stand for the magnitude of the mean changes as following: t = trivial; s = small; m = moderate; l = large; vl = very large. Asterisks indicate the likelihood for the magnitude of the true effect as follows: * possible; ** likely; *** very likely; **** most likely. Letters and asterisks next to the x-axis indicate the magnitude and uncertainty of the mean changes from NC to the QSC. Letters and asterisks above and below the error bars indicate the magnitude and uncertainty of the mean changes from the rest status to the 2^nd^ to 10^th^ repetition and post-test status in NC and QSC. Effects are unclear in the situations without letters or asterisks.

The mean values of each parameter in each repetition in NC and QSC are presented in Figs 1–4. As can be seen from Fig 1, in the 1st -5th and the 9th repetition, the players achieved higher peak power in QSC than in NC, while in the 1st and 5th repetitions, the players achieved higher mean power and total work in QSC than in NC. In the 1st– 3rd and the 5th repetitions, the players used less time to attain peak power in QSC than in NC. The fatigue slope and fatigue rate in the 1st to 4th, 7th and 9th repetitions in QSC were higher than in NC. In NC, compared to the 1st repetition, the players achieved higher peak power in the 3rd, 4th, 7th, 8th, and 10th repetitions, higher mean power and total work in the 2nd repetition, as well as higher fatigue slope and rate of fatigue in the 7th, 8th, and 10th repetition, but needed less time to peak power in the 4th, 7th and 8th repetition. While in QSC, the mean power and total work achieved by the players in the 3rd repetition showed a non-significant difference from the 1st repetition, the fatigue slope and rate of fatigue increased in the 7th repetition compared to the 1st repetition.

According to data from Fig 2 the V’E of players in the 1st and 2nd repetitions and the RER of players in the 1st repetition in QSC were higher than in NC, while the players’ choice reaction time in the 5th repetition in QSC was lower than in NC. Compared to the 1st repetition, the V’E, V’O2 and RER increased in the 2nd to 10th repetitions, the HR and RPE increased in the 3rd to 10th repetitions in NC, while in QSC, the V’E, HR and RPE increased in the 3rd to 10th repetitions, and the RER increased in the 3rd and 4th repetitions. Meanwhile, the choice reaction time in the 7th and 10th repetitions in NC decreased compared to the 1st repetition.

As shown in Fig 3, the players’ PO2 in the post-test status in QSC were higher than in NC. Compared to the 1st repetition, the PCO2 decreased in the 4th, 6th, 8th, 10th repetitions and post-test status in NC and QSC, the PO2 increased in the 4th, 6th, 8th, 10th repetitions and post-test status in NC and increased in the 6th, 8th, 10th repetitions and post-test status in QSC, the PH decreased in the 2nd, 4th, 6th, 8th, 10th repetitions and post-test status in NC and QSC, the LAC and HCT increased in the 2nd, 4th, 6th, 8th, 10th repetitions and post-test status in NC and QSC.

According to Fig 4 the players’ CL levels in the 4nd, 6th repetitions and post-test status in QSC were higher than in NC, while NA+ in the rest status and K+ in the 2nd, 6th and 10th repetitions in QSC were lower than in NC. Compared to the 1st repetition, the concentration of NA+ increased in the 2nd, 4th, 6th, 8th, and 10th repetitions in NC, and increased in the 2nd, 4th, 6th, 8th, 10th repetitions and post-test status in QSC, the concentration of K+ increased in the 2nd, 4th, 6th repetition but decreased in the post-test status in NC and increased in the 2nd, 4th, 6th, and 8th repetitions but decreased in the 10th repetition and the post-test status in QSC, the concentration of Ca2+ increased in the 4th and 6th repetitions in NC and QSC, but decreased in the post-test status in NC, the concentration of Cl- increased in the 2nd, 4th, 6th, and 8^th^ repetitions but decreased in the post-test status in NC and QSC.

## Discussion

The study results contribute to the understanding of effects of different climatic conditions on RME performance, and are particularly relevant for soccer players’ preparation for the 2022 World Cup in Qatar. The main study findings were: 1) The difference in climatic conditions, up to 8 degrees, does not cause too much change in the players’ body response to repeated maximal efforts. The greatest changes were recorded in the power-related variables demonstrating higher maximal power values and shorter time to attain peak power in QSC, with a higher fatigue slope and rate of fatigue rate than in NC. 2) More substantial changes were demonstrated by comparing successive repetitions with the 1^st^ repetition. Considering the players’ body reactions in both examined sets of conditions, it is worth noting that the direction of changes was similar, apart from the power variables. Moreover, in QSC conditions, most of the physiological variables substantially increased from the 3^rd^ repetition and remained up to 10. However, in both sets of conditions, the most significant changes were found in blood-related and electrolytes-related variables, some variables increased and others decreased due to the developing fatigue.

### Comparison between the NC vs QSC conditions

Exercising in warm to hot (25–45°C) environments poses significant challenges to the human regulatory system [47, 48] inhibiting the performance of repeated maximal efforts or sprints in team games or endurance sports [49]. The detrimental effects of environmental temperatures above 30°C, compared with cool conditions, on repeated maximal efforts have been documented in tests using cycle ergometry [50], treadmill [51], and field runs [49, 52]. Considering power-related variables, the studied soccer players performing the protocol in QSC achieved higher RME values in each repetition. A higher ambient temperature also influenced shorter / faster generating power. This is consistent with the results of the most recent study by Chodor et al. [7] which demonstrated similar dependencies. The comparison with other RME protocols is difficult because in many cases the length of the rest break was different, more often shorter than longer. For example, Girard et al. [31] found that the average maximal power (Pmax) was 3.1% higher in HOT (35°C) than in NC (24°C). Moreover, Yamaguchi et al. [53] reported Pmax in HOT (35°C) 3% higher than in NC (20°C). Frikha et al. [54] noted that the higher mean power-output values in HOT (30°C) conditions may be related to the higher increase in core temperature [55]. This increase was shown to be a major factor responsible for improving nerve conduction velocity, enzymatic activities, oxygen delivery to muscles as well as for decreasing muscular viscous resistance [56]. Similarly, an increase in muscle temperature was shown to be responsible for a ~ 4% improvement of muscular leg power for each elevated 1°C [54].

Cardiopulmonary fitness is also often cited as a significant characteristic of success in soccer due to the large distances and continual dynamic movement patterns players are required to perform during a game [57]. Maximal or very high-intensity exercise in the heat causes a relevant impairment in oxygen delivery to the exercising muscles related to cardiac and muscle blood flow decreases [47, 54]. Drust et al. [29] reported lower accumulated oxygen consumption during repeated sprints in HOT (40°C) than in NC (20°C) conditions. Decreased maximal oxygen consumption was previously observed in hyperthermia [58], which was associated with the lowering of cardiac output and mean arterial pressure and their associated effects on skeletal muscle blood flow and oxygen uptake and delivery [47]. Little attention has been paid so far to ventilatory capacity. Studies into ventilatory response to exercise in soccer players were conducted by di Paco et al. [59, 60] but in less specific conditions, e.g. progressive tests, not using the RME protocols. The present study, however, did not reveal many significant changes between the conditions in this group of variables. Périard et al. [61] who studied 6-second repeated maximal efforts in NC (20°C) and HOT (40°C) conditions reported no significant differences in RPE. The completion of the intermittent protocol in HOT conditions was associated with changes in glycogen utilization, blood glucose, noradrenaline, heart rate and RPE, compared with exercise in NC. Some of these physiological changes could potentially lead to alterations in participants’ physiological state prior to the repeated sprints that may impair their ability to perform high intensity exercise [29]. Studies on psychomotor performance, including choice reaction time in heat stress, revealed a deterioration of performance on a central executive task (random movement generation) but not on verbal and spatial recall, and choice reaction time tasks [62]. Central executive tasks are inhibited by heat stress and moving away from the source of the heat and rehydration have some effect on cognition returning to normal [62]. Also, not many changes to these variables were observed in the present study protocol. The only observation worth emphasizing was the substantially shorter CRT in the middle of the protocol, after the fifth repetition in QSC conditions as compared to NC.

The analysis of blood-related variables in the present study revealed no significant differences between the climatic conditions, i.e. the levels of blood-related were very similar in both sets of conditions. Similarly, Yamaguchi et al. [53] found no significant differences in the RSA protocol between NC (20°C) and HOT (35°C) in blood acid-base balance parameters such as LAC, PH, HCO3 and PO2. Furthermore, Mohr et al. [9] when comparing NC (21°C) and HOT (43°C) match conditions noted no significant blood lactate concentration differences in studied players. In the present study these parameters did not differ significantly between QSC and NC either. In fact, Yamaguchi et al. [53] revealed a 15°C difference between HOT and NC, while Mohr et al. [9]–a 22°C difference; however, no difference between climatic conditions was found. It can be concluded that the temperature difference does not modify blood-related variables for short (6-second) repeated maximal efforts. In support of this thesis Drust et al. [29] stated that the concentrations of metabolic parameters were not significantly different between the experimental trials at the end of the intermittent protocol. This would indicate that the changes in performance were not associated with the accumulation of these peripheral fatigue products. Furthermore, Yamaguchi et al. [53] noted that there is still insufficient research on the responses of electrolyte levels between different simulated conditions during repeated maximal efforts. The present study shows that more changes were recorded in the level of electrolytes (in K+ and CL–) comparing QSC and NC. This indicates that players should be alert to regular fluid replenishment, isotonic drinking, and that training staff should control players’ dehydration status. More studies should also be designed to monitor changes in electrolyte concentration in players performing intermittent efforts in team games in different climatic conditions, especially in high temperatures.

### Analysis of trial time between the first and subsequent repetitions

Heat stress is commonly associated with a shorter time to exhaustion [63, 64] or longer trial completion time [61, 65]. There have been, however, few studies regarding the effects of hot ambient conditions on the performance of repeated maximal exercise. In their analysis of even repetitions of a 10-trial protocol Chodor et al. [7] observed that players’ peak power increased in QSC and decreased in NC conditions; however, the changes were non-significant, i.e. the players maintained their power values in the protocol with a 90-second break. In turn, Girard et al. [31] noted a decrease in Pmax from 1 to 10 repetitions, in 6—second repeated sprints, in both NC and HOT conditions, with a similar rate of power decrease. They also registered a higher Fatigue Index in NC (19.7%) than in HOT conditions (16.5%). The present study revealed more significant changes in NC between 1 and 10 repetitions, most likely because the players’ muscle temperature was not high enough to generate power in the 1^st^ repetition. On the other hand, Drust et al. [29] found greater power decreases in HOT than in NC. Aldous et al. [51] claim that performance decrements were accompanied by earlier and greater increases in core temperature under heat stress, exceeding 39.5°C. Similarly, a strong correlation was found between the rate of core temperature rise and heat-induced reductions in performance (r = 0.90) [52]. Girard et al. [49] also indicated that “normo-thermic” individuals may not necessarily demonstrate lower performance levels in hotter climate conditions. Therefore, despite a higher cardiovascular and perceptual strain in the environmental temperature of 40°C vs 24°C in the absence of hyperthermia the performance levels did not differ between the two environments [66]. In Yaicharoen et al. [67] the final values for core temperature may not have been high enough to unduly affect performance in 36°C as compared with 23°C. Furthermore, enhanced short (< 30 s) power output or single-sprint performance resulting from transient heat exposure (rise in muscle temperature) can be attributed to improved muscle contractility. There is also compelling evidence suggesting that poorer intermittent-sprint performance in hotter conditions occurs only when exercise induces marked hyperthermia (core temperature above 39°C) [49]. This was not found in the present study since the applied protocol was shorter than 30 minutes.

Respiratory parameters and ventilatory response can play a key role in qualitative and quantitative evaluation of professional soccer players’ performance [59]. During the performance of all-out 6-second sprints, oxygen consumption increases rapidly at the onset of sprinting [68] and rises in subsequent sprints [69] to the levels that may exceed 70% VO_2_max [70]. In the present study the players obtained similar levels of this variable in both sets of climatic conditions. During intense intermittent exercise the oxygen consumed between sprints has been associated with enhanced PCr restoration [71], which should result in superior power maintenance on subsequent sprints. Also the higher VO_2_ during the sprints should increase the amount of energy available for muscle contraction by supplementing anaerobic energy, affecting the overall sprinting performance. In support of this Hamilton et al. [70] found a relationship between the aerobic response during repeated sprint-recovery intervals and fatigue in peak power (r = -.60) [72]. Therefore, a higher maximal aerobic power is associated with enhanced aerobic contribution and superior power maintenance during repeated supramaximal cycle sprints in female recreational soccer players [72]. However, Aziz et al. [73] as well as others [74] suggest a weak association between aerobic fitness (VO_2_max) and running RSA performance in team-sports players. Then, Kerhervé et al. [20] observed that a significant increase in RPE levelled off after the fifth bout, and that maximum RPE was reached at the 10th bout of exercise. In our research players reported substantially higher RPE in both conditions after 3 repetitions, and also the highest value of this variable was recorded in the final 10th repetition. A similar body response in soccer players was observed following HR measurements in both climatic conditions. As far as choice reaction time is concerned, it is important to emphasize that in sport successful performances strongly depend on the ability to simultaneously meet cognitive and physical demands. It has been established that acute moderate exercise enhances cognitive functions [75–77]. Davranche and McMorris [78] also showed that most of this improvement is due to better discharge synchronization of the motor units, and, to an extent, it is due to greater efficiency of the peripheral sensorial processes. Chmura et al. [79] showed an inverted-U effect during the performance of a choice reaction time test following the epinephrine (E) and norepinephrine (NE) thresholds being faster than at rest and during maximal intensity exercise. However, McMorris et al. [80] demonstrated a significant improvement at the E threshold and during exercise at maximum power output (:W max). Whereas, McMorris et al. [81] demonstrated no significant effect of exercise. The research results in this regard remain contradictory. Our study confirms the two-phase course of changes in QCS conditions. After exceeding the anaerobic threshold the fastest response occurs, however, a further increase in fatigue reduces psychomotor performance. This is one of the most interesting observations in the entire experiment, which requires further research. Recent studies have examined the effect that undertaking a cognitively fatiguing task for ≤ 90 min has on subsequent physical performance. Cognitive fatigue is claimed to affect subsequent physical performance by inducing energy depletion in the brain, depletion of brain catecholamine neurotransmitters, or changes in motivation [82].

One of the most significant training stimuli for maximizing the effects of repeated maximal exercise is also metabolite (lactate and hydrogen ion) accumulation in working muscles [83]. A single bout of repeated maximal efforts can decidedly increase muscle lactate content and decrease muscle glycogen content [84]. With regard to the blood-related variables in the present study, the PH was shown to decrease and LAC to increase significantly in subsequent maximal exercises in both sets of climatic conditions. The magnitude of these changes was considerable, and their real effect most likely. Since metabolite production and clearance are both closely associated with local blood flow in surrounding muscle tissues, restricted blood flow would enhance metabolite accumulation [85]. The attenuated power output during sprint exercise may be associated with the ability of the neuromuscular system to produce maximal force [86] and/or a relationship between force and velocity during repeated sprints [85]. It had been demonstrated that exercise in the heat leads to greater reliance on muscle glycogen, anaerobic metabolism, and muscle and blood lactate accumulation [87], which generate fatigue and a decline in force production. Fatigue development during high-intensity intermittent exercise may also be caused by a complex interplay between intra and extracellular levels and gradients of K+, Na+, Cl–, H+ and Mg+ [88, 89]. Although contentious [90], critical rises in hydrogen cation (H+) accumulation can be linked to a reduction in the release and uptake of calcium ions (Ca2+) from the sarcoplasmic reticulum [91], disruption of glycolytic pathway key enzymes [92], and lowered muscle excitability and action potentials by decreasing strong ion difference [93]. In turn, this may hamper subsequent performance by reducing the capability for muscle force production [94, 95]. When analyzing the level of selected electrolytes in the repeated maximal effort protocol, many meaningful changes were observed, which is not surprising. This indicates that short-term but repeated efforts have an impact on the level of these variables. These variables should be monitored over time/during a longer protocol, and we should be aware that their disturbances will reduce exercise capacity, especially accompanied by high temperatures.

One limitation of this study was that it was conducted in a climate test chamber, i.e. in a non-specific environment for soccer players. The exercise tests were also performed on a cycle ergometer (not a soccer-specific exercise) without a game-related stressor. Moreover, despite the examination of 4 different groups of parameters, such as power-related, physiological, psychomotor, blood-related and electrolytes-related variables (22 parameters in total), the internal body temperature was not taken into account. The study conclusions should therefore be used with caution. Further research should involve the examination of body responses in players of different ages and at different levels of training experience, in conditions typical for soccer: on a grass pitch, with plenty of running exercises, and with rest breaks of different length. Such tests could be carried out under a special canopy providing specific climatic conditions within it. Prospective studies in varied climatic conditions are necessary to provide a more in-depth analysis of effects of heat stress on repeated maximal exercise performance. It is also suggested to consider the measurement of muscle core temperature, players’ hydration status—especially in longer study protocols—and the WBGT index, which involves more components related to weather conditions.

## Conclusion

The results of the present study contribute to a better understanding of the impact of different climatic conditions on soccer players’ performance, with an emphasis on a broad set of variables determining the different reactions of players performing RME. It has been shown that semi-professional soccer players, by performing a protocol of repeated maximal efforts on a cyclo-ergometer in thermoneutral conditions and simulated conditions of the FIFA World Cup Qatar 2022, demonstrated a greater power output ability in higher temperatures. Furthermore, the 8°C temperature difference between the sets of climatic conditions did not significantly affect the players’ physical and physiological responses.

More changes were observed in blood-related and electrolyte-related variables in subsequent repetitions, where increasing fatigue was recorded in players performing the study protocol. However, their responses were similar to each other in both simulated sets of conditions. Nevertheless, it should be remembered that at higher temperatures, players will start to feel fatigue faster and the decrease in their performance will be greater. To obtain more significant changes, the conditions would have to be more different, or the protocol would have to be extended. Therefore, the postponement of the World Cup to November and December will result in players not experiencing significant drops in performance, as if they were playing in June and July.

Repeated sprints are a potent and time-efficient training strategy, effective in developing acceleration, speed, explosive leg-power, aerobic power and high-intensity running performance—all of which are crucial to team-sport performance [15]. The use of cycloergometer tests provides the direct possibility of registering soccer players’ mechanical parameters and reactions in simulated climatic conditions, which cannot be assessed on the pitch. The results of this study indicate that the ambient temperature in which training sessions are conducted and matches played must be considered in order to predict players’ exercise capacity and to adopt an appropriate match strategy.

## Practical application

The search for optimal solutions for soccer players preparing for top-level tournaments is important. The climate conditions in which matches are played must be accounted for in planning preparations for future World Cup tournaments, especially those in hotter countries. The results of this study can be used in the design of training programs aimed to improve players’ physiological adaptations by simulating soccer-specific playing conditions for developing anaerobic capacity in repeated maximal exercise. It is recommended that different protocols of repeated maximal exercise in high-temperature conditions should be performed to increase tolerance to this type of effort. Since the weather is an external and non-modifiable factor, heat preparedness efforts should focus on modifiable and internal personal factors, such as acclimatization, hydration, nutrition, pre-cooling, as well as on provision of breaks when needed, and lowering temperatures in warm-up and competition areas [96]. It is recommended also that the national team players should arrive in Qatar as soon as possible after the end of their respective league seasons. It is also advised to organize a recovery camp to ensure the players’ adaptation to climate conditions in Qatar, and then a direct preparation session for the first match of the World Cup. This will enable a delayed onset of subjective feeling of fatigue, smaller changes in body’s acid-base balance, and a minimum power loss in the final phase of the game. These findings are of significant relevance for the upcoming 2022 World Cup in Qatar as well as any other match locations with customary high ambient temperatures.

## Supporting information

S1 Data (XLSX)
